# Investigating alexithymia in autism: A systematic review and meta-analysis

**DOI:** 10.1016/j.eurpsy.2018.09.004

**Published:** 2019-01

**Authors:** Emma Kinnaird, Catherine Stewart, Kate Tchanturia

**Affiliations:** a King’s College London, London, Institute of Psychiatry, Psychology and Neuroscience, Department of Psychological Medicine, UK; b South London and Maudsley NHS Foundation Trust, London, UK; c Illia State University, Tbilisi, Georgia

**Keywords:** Autism, Psychometry and assessments in psychiatry (tests)

## Abstract

**Background:**

New research suggests that, rather than representing a core feature of autism spectrum disorder (ASD), emotional processing difficulties reflect co-occurring alexithymia. Autistic individuals with alexithymia could therefore represent a specific subgroup of autism who may benefit from tailored interventions. The aim of this systematic review and meta-analysis was to explore the nature and prevalence of alexithymia in autism using the Toronto Alexithymia Scale (TAS).

**Methods:**

Online scientific databases were searched systematically for studies on ASD popu lations using the TAS. Meta-analyses were performed to evaluate differences in scores between the ASD and neurotypical groups, and to determine the prevalence of alexithymia in these populations.

**Results:**

15 articles comparing autistic and neurotypical (NT) groups were identified. Autistic people scored significantly higher on all scores compared to the NT group. There was also a higher prevalence of alexithymia in the ASD group (49.93% compared to 4.89%), with a significantly increased risk of alexithymia in autistic participants.

**Conclusions:**

This review highlights that alexithymia is common, rather than universal, in ASD, supporting a growing body of evidence that co-occurring autism and alexithymia represents a specific subgroup in the ASD population that may have specific clinical needs. More research is needed to understand the nature and implications of co-occurring ASD and alexithymia.

## 1 Introduction

Autism Spectrum Disorder (ASD) is a neurodevelopmental disorder characterised by difficulties in social communication and interaction, and restricted or repetitive patterns of behaviour or interests [1]. However, ASD has also been associated with difficulties in emotion processing, in particular problems with recognising emotions in others [2,3].

Nonetheless, findings on emotion processing in ASD have been inconsistent [4,5], leading to suggestions these difficulties may not represent a core feature. Rather, it has been suggested that these the problems with emotion processing often observed in ASD instead stem from co-occurring alexithymia [[6], [7], [8]]. First described in the 1970s, alexithymia refers to difficulties in recognising and distinguishing between different emotions and bodily sensations, difficulties in expressing emotions, a lack of imagination or fantasy life, and thoughts focused on external rather than internal experience [9].

Significantly, alexithymia is thought to be heightened in autistic people compared to the general population [[10], [11], [12]]. An increasing body of empirical research supports the hypothesis that emotion processing difficulties in ASD are in fact driven by alexithymia. Studies controlling for both alexithymia and autism have found that alexithymia, rather than autism, predict difficulties in facial, vocal and musical emotion recognition [8,13,14]. Furthermore, imaging research suggests that empathetic brain activity in response to the pain of others is predicted by alexithymia, not autism [15].

There are a number of potential mechanisms which could underpin this relationship between ASD and alexithymia. A meta-analysis of neuroimaging studies suggests that alexithymia may be associated with reduced activation in a number of brain areas associated with emotion processing, specifically the amygdala, mirror neuron system related brain regions, the dorsomedial prefrontal cortex, and the right insula and precuneus [16]. Although more research is needed on the potential links to alexithymia in this population, autism is known to be associated with atypical neural connectivity, including in the amygdala and insula [[17], [18], [19]]. Consequently, it has been proposed that both autism and alexythmia may both be associated with a genetic vulnerability to atypical brain connectivity that can manifest as either “pure” autism, “pure” alexithymia, or co-occurring autism and alexithymia, depending on the exact networks affected [6].

Alternatively, another potential shared mechanism between alexithymia and autism could be that of mentalizing: both constructs are known to be associated with mentalizing difficulties [20,21]. However, an imaging study found that difficulties in emotional awareness in autistic people was not associated with variations of brain activity in the mentalizing system [3]. Rather, these difficulties were associated with reduced activation in the anterior insula, an area thought to be key in enabling the conscious representation of feelings, and highly correlated in this study with self-rated alexithymia. Consequently, the authors concluded that their findings supported “decoupling” models of alexithymia, where the physiological arousal induced by an emotional state is not integrated with conscious awareness of this arousal. Significantly, this could represent a key shared mechanism between autism and alexithymia, reflecting research suggesting that there is a disruption between how autistic individuals subjectively experience their emotions, and their physiological emotional arousal [20]. Consistent with this hypothesis are findings from a recent study finding that self-reported alexithymia was associated with reduced skin conductance, suggesting reduced emotional experience, and disruption between subjectively and objectively reported measures of emotional arousal, supporting the role of decoupling in alexithymia and autism [22].

However, not all autistic people have alexithymia, with a recent study finding a prevalence rate of 55% in autistic adolescents [23]. Consequently, Bird & Cook [6] have proposed the “alexithymia hypothesis” of ASD: that the emotion processing difficulties seen in ASD stem from co-occurring alexithymia, rather than representing a core feature. In line with this hypothesis, research has found that alexithymia, and not ASD, is predictive of problems with emotion processing [8].

This suggests that individuals with both alexithymia and ASD represent a distinct subgroup of autistic people who may benefit from interventions that could help manage these emotional processing difficulties [24]. Understanding the potential co-occurrence of alexithymia in autism is vital for both clinical and research purposes. Alexithymia may be associated with additional difficulties for autistic people, with this same adolescent study finding that individuals with both ASD and alexithymia experienced higher levels of anxiety and emotional difficulties compared to those with ASD only [23]. Moreover, autistic people are known to be at heightened risk for a number of mental health problems [25], and alexithymia is associated with poorer outcomes for psychotherapeutic treatment [26]. Therefore, individuals with co-occurring autism and alexithymia may benefit from targeted interventions, such as training in identifying and communicating feelings, or mindfulness exercises ([27] [28];). The alexithymia hypothesis also has significant implications for future research on emotion processing in ASD, suggesting that it may be necessary for future studies on this and related areas to control for alexithymia in their design and analysis [8].

At present, the measurement of alexithymia primarily relies on self-report measures requiring participants to reflect on their difficulties with identifying their emotions, which, when measuring a construct associated with problems reflecting on emotion identification, has been noted as a counter-intuitive approach (Gaigg, Cornell & Bird., 2016). However, the above study examining the variance between self-reported subjective emotional arousal, and objective arousal as measured by skin conductance, in autistic and control participants, found good correlations between self-report alexithymia measures and this objective, experimental method (Gaigg, Cornell & Bird., 2016). In 2005, Berthoz & Hill confirmed that the 20-item Toronto Alexithymia Scale (TAS-20) can be used to reliably identify alexithymia in an ASD population, with the measure demonstrating good convergent validity [10]. There is also a longer, 26 item version of this measure known as the TAS-26. The TAS-20 presents participants with 20 items, to which they rate their level of agreement on a Likert Scale from 1 (strongly disagree) to 5 (strongly agree) [29]. This yields an overall total score, with a score of 61 and above indicating high levels of alexithymia. The TAS-20 additionally generates scores for three subscales measuring difficulty identifying feelings (DIF); difficulty describing feelings (DDF) and externally-oriented thinking (EOT). Therefore, the TAS may be used to assess the presence of alexithymia in autistic people. However, the TAS does have some key weaknesses: it does not measure the fantasy aspect of alexithymia, and the EOT scale may lack reliability [30]. Consequently, it has been recommended that the TAS should not be the only measure used to evaluate alexithymia. Commonly used alternative measures include the Bermond-Vorst Alexithymia Questionnaire (BVAQ), which does include the fantasy construct [31].

As well as giving insight into the prevalence of alexithymia in ASD, a systematic review is necessary to illuminate the use of the TAS in ASD, including consideration of confounding variables and the utilisation of additional measures. Therefore, this review aimed to synthesise the literature on the use of the TAS in autistic people by using a meta-analysis to explore differences between ASD and NT groups on alexithymia scores. It is predicted that autistic people will experience heightened levels of alexithymia compared to NT groups, but that not all autistic people will experience alexithymia.

## 2 Methods

The study was conducted according to PRISMA guidelines [32].

### 2.1 Eligibility

This review included studies using either the TAS-20 or TAS-26 with both ASD and neurotypical (NT) populations. Inclusion criteria were 1) full text available in English, 3) published in a peer reviewed journal, 3) reporting a comparison of total mean TAS scores for both populations with standard deviations. Studies which used the TAS to match ASD and NT groups for alexithymia, rather than comparison, were also excluded.

### 2.2 Information sources and search

The databases PsychInfo, Scopus, Pubmed and Web of Science were searched for papers up to and including January 2018. The search terms were autis* and alexithymia, and Toronto Alexithymia Scale. “Or” Bermond-Vorst Alexithymia Questionnaire (BVAQ) was additionally incorporated as a search term in order to highlight papers utilising this common additional measure for alexithymia in this population.

### 2.3 Selection

**Fig. 1:**
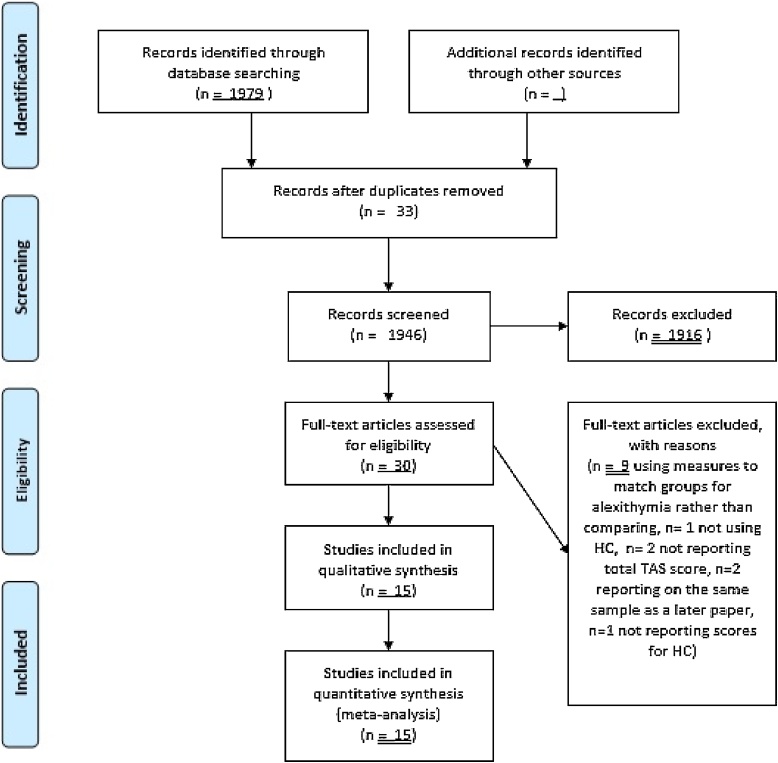
PRISMA diagram of selection process.

The selection process is summarised in Fig. 1. Following the exclusion of duplications, the titles of papers were screened for relevance. Abstracts of titles which appeared to potentially meet the criteria were then screened. Full texts were retrieved if the abstract indicated that inclusion criteria were met, or if there was not sufficient information in the abstract to warrant a decision. Full texts were reviewed, with any that did not meet the inclusion criteria excluded with reasons given.

### 2.4 Data collection and items

The following data was extracted from each paper: gender and age of participants, how ASD and NT groups were matched, TAS version, use of additional alexithymia measures, recruitment source, how ASD was diagnosed, comorbidities assessed, mean total TAS scores with subscale scores if reported, and number of participants in each group scoring above cut-off for alexithymia on the TAS-20 (defined as >61).

### 2.5 Risk of bias across studies

Risk of bias across studies was assessed visually using funnel plots, plotting standard error against standard mean difference (effect size). The Duval and Tweedie nonparametric "trim and fill" method was also implemented using the *metatrim* command in Stata15 to assess publication bias by estimating the number and outcomes of missing studies. Between-study heterogeneity was measured using the Cochrane Q test.

### 2.6 Risk of bias in individual studies

Risk of bias for each study was assessed by evaluating the quality of each study using the Clinical Appraisal Skills Programme Checklist for case-control studies, in line with previous research in this area [33]. The tool uses 11 questions to assess study quality, including whether potential confounding variables were accounted for in analysis or study design, and how participants were recruited. An overall quality rating was calculated by dividing several questions into sub-questions with a score of 1 for every “yes” response, giving a maximum quality rating of 17.

### 2.7 Summary measure

The principle measure used for meta-analysis was the difference between ASD and NT groups on mean scores and standard deviations on the TAS total score, and, if reported, subscale scores. Where studies had subdivided their ASD and NT groups into smaller sub-groups, such as by gender, these scores were combined into overall ASD and NT means using the *combine* command in Stata15. For the prevalence and risk ratio analysis, the principle measure was the number of autistic people scoring as alexithymic on the TAS compared to NT. Due to the requirements of the analysis, where studies only reported percentages of participants scoring as alexithymic in each group this was converted using sample size information into the number of participants and, if necessary, rounded to the nearest whole number.

### 2.8 Synthesis of data

The meta-analysis brought together all studies reporting mean TAS scores, and standard deviations for ASD and NT groups. Standardised mean differences were used to compare studies as some studies used the TAS-26, whilst other used the shorter TAS-20. The meta-analysis used a random effects model. This model accounts for between study heterogeneity and adjusts the study weights accordingly.

### 2.9 Statistical analyses

Meta-analyses were conducted using Review Manager 5.3, with some additional functions performed using Stata15 [34]. Comparison of TAS total and subscale scores between groups was calculated by using Cohen’s *d* to estimate effect sizes for each study, interpreted as small (0.2), medium (0.5) or large (0.8). A positive effect size indicated that the ASD group scored higher on the TAS mean scores compared to the NT group. Following initial analyses, meta-regression was performed in Stata15 using the *metreg* command to analyse associations between overall TAS score, mean age, and age difference between ASD and NT groups. A weighted prevalence rate was calculated by weighting the mean percentage of participants scoring above the cut-off for each group according to the number of participants in each study. The Cochrane-Mantel-Haenszel random effects estimate method was used to calculate the risk ratio of scoring above the TAS cut-off for alexithymia in ASD compared to NT groups.

## 3 Results

### 3.1 Study selection

The systematic review identified 17 studies as eligible for inclusion. Three studies reported on an overlapping sample, with data from some participants being used in multiple studies [10,12,35]. For the purposes of this analysis, the most recent paper only [35] was used as it represented the largest sample. Therefore, a total of 15 studies were included in this systematic review.

### 3.2 Study characteristics

The 15 studies evaluated in this systematic review are summarised in Table 1. 12 studies used the TAS-20, whilst 3 studies used the longer TAS-26. 11 studies reported subscale scores, and 9 studies reported how many individuals in each group scored above the cut-off for alexithymia.

**Table 1:** Summary of studies included in systematic review.

Year	Author	Group	N	Gender (male (female))	Mean Age (SD)	NTs Matched By	TAS Version	Additional measure?	Recruitment Source	ASD Diagnostic Tool	Comorbidities assessed	TAS Total Score	N (%) Above cut-off (>61)	TASF1 DIF	TASF2 DDF	TASF3 EOT	Quality Score
2017	Arellano et al.	ASD NT	14 21	14 (0) 14 (0)	15.33 (0.99) 15.64 (1.15)	Age, IQ	TAS-26	BVAQ-AB				43.79 (9.78) 37.15 (7.32)		14.07 (6.03) 10.50 (3.10)	14.14 (5.20) 11.40 (4.01)	15.57 (3.20) 15.25 (3.99)	9
2017	Murray et al.**	ASD NT	19 20	20 (0) 19 (1)	30.60 (6.52) 30.65 (6.27)	Age, gender, verbal ability	TAS-20		ASD diagnostic service	ICD-10		61.58 (10.07) 46.60 (11.10)	11 (52.6%) 4 (20%)	20.58 (5.98) 15.60 (6.02)	17.95 (3.46) 12.95 (5.27)	23.05 (4.48) 18.05 (4.44)	13
2017	Schaller & Rauh.	ASD NT	23 22	23 (0) 22 (0)	15.72 (1.25) 15.85 (0.97)	Gender, age, nonverbal intelligence	TAS-26		University project databases	ADOS, ADI-R		45.32 (7.63) 38.36 (6.73)		14.64 (5.01) 10.82 (3.43)	14.96 (3.51) 11.6 (3.50)	15.65 (3.26) 15.86 (3.37)	13
2016	Hoffmann et al.	ASD NT	25 25	25 (0) 25 (0)	32.6 (8.5) 32.4 (8.5)	IQ, gender	TAS-26		Outpatient clinic, referral from clinician	ADOS, ADI-R		54.2 (10.0) 37.4 (7.8)					13
2016	Milosavljevic et al.*	ASD NT	56 32	54 (2) 32 (0)	15.45 (0.48) 15.5 (0.57)	Age, gender	TAS-20		Prior autism prevalence study cohort	ADOS, ADI-R	Depression, anxiety	53.11 (11.64) 45.63 (11.64)	31 (55%) 5 (16%)	16.34 (6.28) 12.03 (5.45)	13.63 (4.34) 10.97 (4.53)	23.14 (4.23) 22.63 (3.51)	14
2016	Patil et al.	ASD NT	15 16		37.35 (13.02) 32.03 (9.44)	Age, gender, education	TAS-20		ASD organisations, internet communities	ICD-10	Depression	53.60 (8.63) 34.75 (3.96)	7 (47%) 0 (0%)	20.13 (5.01) 9.63 (1.86)	20.20 (2.51) 11.38 (2.19)	13.27 (3.37) 13.75 (2.52)	12
2015	Krach et al.	ASD NT	16 16	16 (0) 16 (0)	21.5 24.3	Age, gender, verbal IQ	TAS-20			ICD-10, ADOS, ADI-R		55.53 (14.3) 44.93 (10.02)					11
2013	Berthoz et al.	ASD NT	38 47	63% male 62% male	35.5 (13.3) 33.7 (11.7)	Age & gender accounted for in analysis	TAS-20	BVAQ-B	Support groups and community centres	DSM-IV	Depression, anxiety	61.4 (12.2) 40.0 (9.2)	21 (55.3%) 1 (2.1%)	21.6 (6.7) 13.7 (6.4)	18.1 (4.2) 11.5 (5.2)	21.7 (5.1) 17.7 (4.8)	15
2013	Schneider et al.*	ASD NT	28 27	15 (13) 15 (12)	31.39 (8.97) 31.42 (9.08)	Gender, age, education	TAS-20		Inpatient and outpatient facilities			59.88 (11.61) 39.48 (9.96)					13
2012	Heaton et al.	ASD NT	20 20	15 (5) 15 (5)	33.70 (12.77) 33.60 (12.06)	Age, IQ, gender	TAS-20		National Autistic Society website, social groups, community centres	DSM		60.70 (15.47) 36.10 (8.85)	9 (45%) 0 (0%)				13
2012	Samson et al.	ASD NT	27 27	11 (16) 11 (16)	33.56 (12.82) 35.22 (12.82)	Gender, age, educational level completed	TAS-20		Participants in previous studies			61.41 (10.85) 42.70 (10.35)	17 (63%) 0 (0%)	23.63 (4.76) 14.52 (5.15)	18.19 (4.24) 11.22 (3.66)	19.59 (4.67) 16.96 (3.95)	13
2008	Katsyri et al.	ASD NT	20 20	13 (7) 13 (7)	32 (10) 31 (8)	Age, gender	TAS-20		Neurology hospital, ASD clinic	ICD-10, DSM-IV, ADOS, ADI-R		55 (12) 36 (6)	7 (35%) 0 (0%)	21 (5) 11 (3)	16 (6) 9 (2)	18 (5) 15 (5)	13
2008	Silani et al.	ASD NT	15 15	13 (2) 13 (2)	36.6 (11.7) 33.7 (10.3)	Age, IQ, gender	TAS-20	BVAQ-B		DSM-IV, ADOS		55.6 (9.7) 43.7 (12.7)	5 (33.3%) 1 (0.1%)	18.5 (6.5) 14.3 (5.3)	17.2 (4.2) 11.8 (4.4)	19.9 (3.1) 17.6 (5.3)	12
2007	Lombardo et al.	ASD NT	30 30	23 (7) 23 (7)	29.13 (7.40) 29.93 (7.83)	Age, gender	TAS-20			DSM-IV, ICD-10		58.37 (14.19) 41.97 (9.19)		20.03 (6.70) 13.50 (4.82)	16.87 (5.62) 11.10 (4.85)	21.47 (4.90) 17.37 (4.16)	11
2004	Tani et al.	ASD NT	20 10	14 (6) 7 (3)	27.2 (7.3) 26.5 (8.1)	Age, gender, intelligence, body mass index (BMI)	TAS-20		Autism clinic	DSM-IV, Autism Spectrum Screening Questionnaire, SCID	Depression	54.2 (12.4) 34.5 (5.1)	8 (40%) 0 (0%)	19.7 (5.8) 10.3 (3.3)	15.6 (5.1) 8.7 (1.9)	18.9 (5.3) 15.5 (3.8)	14

TASF1: difficulties in identifying feelings.TASF2: difficulty in describing feelings.TASF3: externally oriented thinking.

* Data from separate groups collected into ASD and NT using Stata15.

** Percentage above cut-off converted into number of participants.

Quality of individual studies was generally high: all studies reported mean age, and all studies aside from Patil et al (2016) reported participant gender. Additionally, all studies matched ASD and NT groups on at least some characteristics, most commonly gender and age. The lowest scoring study on the quality appraisal was Arellano et al. [36], due to a lack of information on how participants were recruited, and how ASD diagnoses were defined or confirmed. The highest scoring study on the quality appraisal was Berthoz et al. [35], primarily due to their consideration of confounding factors in the analysis. Only a minority of studies assessed the potential confounding factors of anxiety or depression, and only one study accounted for these factors in their analysis. Berthoz et al. [35] presented group comparisons between ASD and NT for both levels of alexithymia unadjusted for confounding factors, and alexithymia adjusted for depression, as measured by the Beck Depression Inventory [37], and anxiety measured using the State Trait Anxiety Inventory Form Y [38]. Differences between the ASD and NT group on both the TAS total score and all subscale scores remained significant following the control for anxiety and depression.

Despite previous recommendations that the TAS should not be used in isolation, only three studies used an additional measure to assess the presence of alexithymia [30]. In all cases, this was a version of the Bermond-Vorst Alexithymia Questionnaire. Two studies found a correlation between the BVAQ and TAS total scores [35,36], whilst the third study did not report this information [3]. Further information on study quality appraisals may be found in the appendix.

### 3.3 Meta-analysis

#### 3.3.1 Risk of Bias

The funnel plot for total TAS scores is shown in Fig. 1. The funnel plot suggested a potential publication bias due to its asymmetrical appearance, with a small gap in the lower left hand corner of the graph suggesting that smaller effect size studies may be missing from this review of published papers. However, further analysis using the trim and fill method indicated that no studies were missing, with estimated effect sizes remaining unchanged.

#### 3.3.2 TAS score comparison

**Fig. 2:**
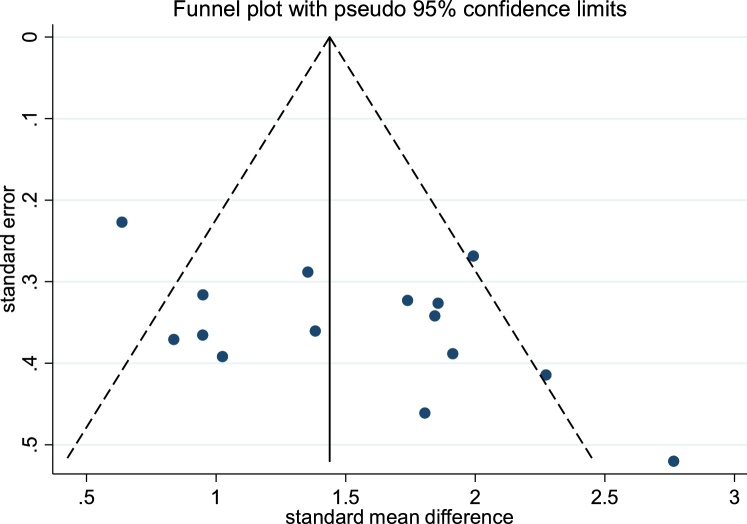
Funnel plot of studies included in the meta-analysis for the assessment of publication bias.

**Fig. 3:**
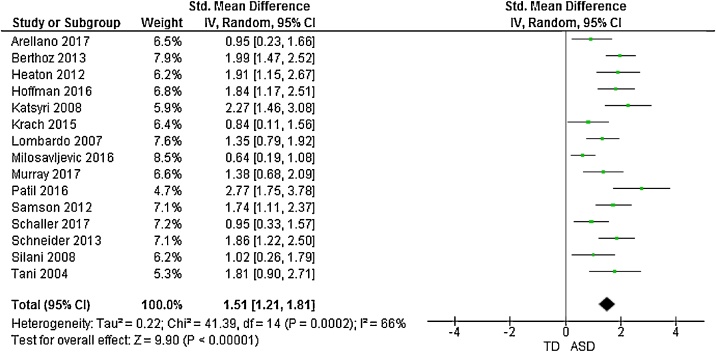
Forest plot of standardized mean effect size for differences between ASD and NT groups on total TAS scores.

**Fig. 4:**
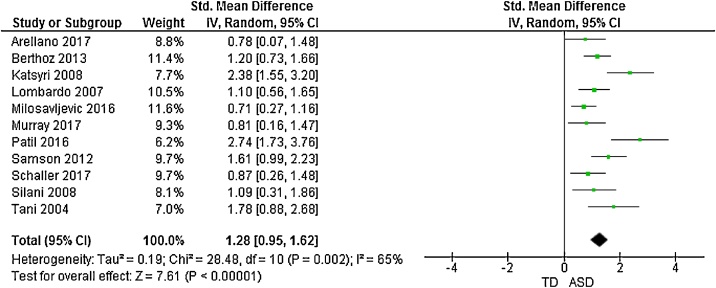
Forest plot of standardized mean effect size for differences between ASD and NT groups on DIF scores.

**Fig. 5:**
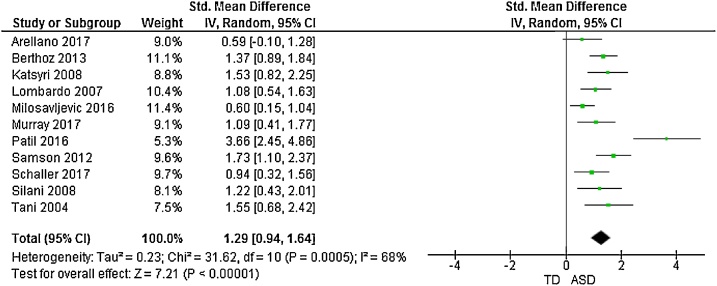
Forest plot of standardized mean effect size for differences between ASD and NT groups on DDF scores.

**Fig. 6:**
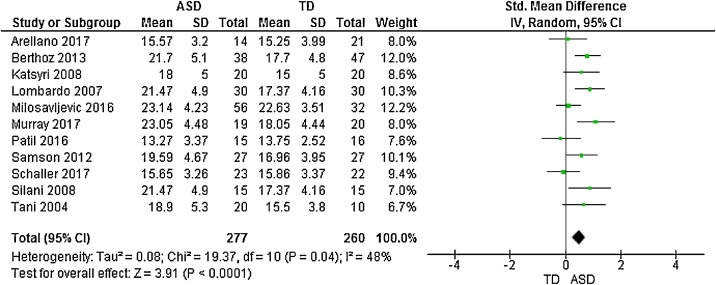
Forest plot of standardized mean effect size for differences between ASD and NT groups on EOT scores.

The forest plots of studies comparing groups on total and subscale TAS scores are displayed in Fig. 2, Fig. 3, Fig. 4, Fig. 5, Fig. 6. Data were extracted from 15 studies giving an overall sample size of 366 autistic people, and 348 N T individuals. The random effects analysis revealed a significant difference between the groups with a large effect size (*d* = 1.51, (95% CI 1.21, 1.81), *Z* = 9.90, *p* <  0.001).

11 studies additionally presented mean scores for ASD and NT groups on the TAS subscales: difficulty identifying feelings (DIF); difficulty describing feelings (DDF) and externally-oriented thinking (EOT). This produced an overall sample size of 292 autistic people, and 275 N T individuals. The random effects analysis revealed a significant difference with a large effect size between the groups for the DIF subscale (*d* = 1.28, (95% CI 0.96, 1.60), *Z* = 7.81, *p* <  0.001) and DDF subscale (*d* = 1.29, (95% CI 0.94, 1.64), *Z* = 7.21, *p* <  0.001). There was also a significant difference between groups on the EOT subscale, with a medium effect size (*d* = 0.50, (95% CI 0.25, 0.75), *Z* = 3.91, *p* <  0.001).

Results suggested significant heterogenity in the overall TAS score meta-analysis (*X^2^* = 41.39, *p* <  0.001). Consequently, a meta-regression was performed to analyse associations between overall TAS score, mean age, and age difference between clinical and control groups. There was a significant effect of mean age on outcome, (b = 0.05 (95% CI 0.03, 0.08), t = 4.51, *p* <  0.001), but no signicant effect for age difference between groups (b = 0.04 (95% CI -0.11, 0.18), t = 0.56, p = 0.586).

#### 3.3.3 Alexithymia prevalence and risk ratio

9 studies used previously established cut-off scores to categorise participants as alexithymic or non-alexithymic, with a TAS-20 score of 61–100 indicating alexithymia [29]. In these papers, prevalence rates of alexithymia in the ASD groups ranged from 33.3% to 63%, with a mean weighted prevalence rate of 49.93%. Prevalence rates in the NT groups ranged from 0% to 20%, with a mean weighted prevalence rate of 4.89%.

**Fig. 7:**
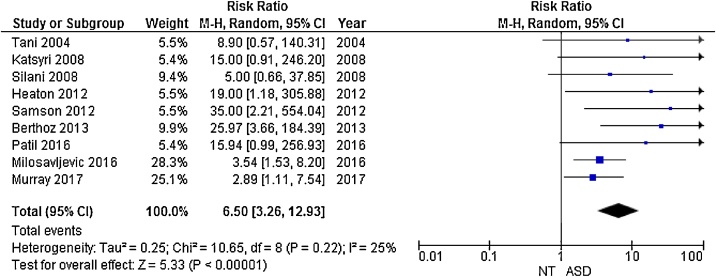
Forest plot of relative risk of scoring above TAS cut-off for ASD and NT groups with confidence intervals.

The Cochran-Mantel-Haenszel random effects analysis revealed an overall risk ratio of 6.50 (95% CI 3.26–12.93, *p* < 0.001) for scoring above the cut-off for alexithymia in autistic people compared to NT (Fig. 7), suggesting a significantly increased risk of alexithymia in the ASD group.

## 4 Discussion

Research suggests that, far from being a core feature of ASD, emotional processing difficulties instead represent a sub-group with co-occurring alexithymia who may have unique needs, particularly surrounding mental health vulnerability and treatment. However, current estimates of the prevalence of alexithymia in ASD vary, with this paper finding estimates between 33.3% and 63%. This was the first systematic review and meta-analysis aimed at exploring alexithymia in ASD using the self –report TAS, a commonly used measure in this field. 15 studies were examined, representing a total of 366 autistic people, and 348 N T individuals. The findings of the meta-analysis suggest that significant differences exist between ASD and NT groups on both total and subscale scores of the TAS, with ASD groups scoring higher on the TAS with medium to large effect sizes. This confirms that autistic people are more likely to experience higher levels of alexithymia compared to their NT counterparts. Furthermore, there was a higher prevalence of alexithymia in the ASD (49.93%) compared to the NT group (4.89%), indicating that alexithymia is common, although not universal, in autistic people. This supports the hypothesis suggesting that an alexithymic subgroup does indeed exist in ASD, and that up to 50% of autistic individuals may be vulnerable to the emotional processing difficulties, and heightened mental health problems, associated with elevated alexithymia [24].

Consistent with previous research in this area, the TAS was found to discriminate between autistic people and NT, with autistic people scoring higher on both total and subscale scores compared to NT. This included on the EOT subscale: research into the use of the TAS in different clinical populations, such as eating disorders, has suggested that the EOT may not discriminate between cases and controls as successfully as the other subscales [33], and the reliability of the subscale across different populations has been questioned [30,39,40]. However, in the present study autistic people were found to score higher on the EOT subscale, albeit with only a medium effect size compared to the large effect sizes exhibited by the total, DIF and DDF subscale scores. This provides further support for the suitability of the TAS, including the subscale scores, in ASD research.

Nonetheless, this review highlighted a number of methodological issues in the application of the TAS in this field. Firstly, the TAS has been criticised previously for not capturing the whole of the alexithymia construct, including the absence of items measuring fantazing or emotionalizing [41]. Consequently, it has been suggested that the TAS should be used together with other measures when exploring alexithymia in ASD, such as the BVAQ [31]. Moreover, an informant based measure such as the Observer Alexithymia Scale may be particularly useful in analysing the presence of alexithymia as the very nature of alexithymia, limiting an individual’s ability to reflect on their own emotions, may additionally inhibit their ability to complete self-report instruments on the subject [42]. However, in this study only three studies used an additional measure- the BVAQ- to assess the presence of alexithymia.

Furthermore, the meta-analysis identified high levels of heterogenity across studies. That mean age was found to impact alexithymia scores in this study is consistent with research suggesting that increasing age is strongly associated with higher levels of alexithymia in a non-clinical population [43]. However, there are a number of additional factors that may have accounted for this heterogenity not captured in the methodology used by the studies in this review. In particular, alexithymia is closely related to depression and anxiety, and both of these conditions are known to be common comorbidities in autistic people [[44], [45], [46], [47]]. Despite this, only one study accounted for depression and anxiety levels in its comparison of TAS scores between ASD and NT groups, finding that differences between the groups reduced but remained significant [35]. This suggests that anxiety and depression may indeed be important confounders when analysing alexithymia in ASD, and highlights the need for future research to consider these variables.

Nonetheless, that only one study considered for the role of confounding factors when analysing alexithymia differences between ASD and NT groups may reflect that, in the majority of studies assessed, alexithymia was not the main focus of the research. Rather, the TAS itself was being used to account for alexithymia as a potentially confounding factor in the area under investigation, such as sleep [48] or social cognition [49]. With an increasing interest in whether individuals with both alexithymia and ASD represent a distinct ASD subtype, any future research using the TAS to explore the alexithymia construct in ASD should address the methodological issues raised in this review, including the use of additional measures and accounting for the significance of potential confounders in analysis [8,24].

The findings from the current review contribute towards the wider literature on alexithymia and related difficulties in ASD. That autistic people were found to score higher on the Difficulty Describing Feelings (DDF) and Difficulty Identifying Feelings (DIF) subscales is consistent with research documenting that the difficulties with recognising, identifying and describing emotions characteristic of alexithymia are also known to be present in ASD [50]. Autistic people are more likely than NT to claim not to feel any emotion, exhibit poorer emotion recognition, have a poorer memory for emotionally significant information, spontaneously mention emotion in conversation, and direct fewer attentional resources towards emotional stimuli [[51], [52], [53], [54]]. Nonetheless, reviews of the available evidence strongly suggest that these difficulties are not unequivocal across ASD, with studies indicating that autistic people are generally able to perceive and identify simple emotions [50,55].

That the symptoms of alexithymia may be a sometimes co-occurring, but not core feature of ASD is supported by the findings of this review. Whilst the meta-analysis found that autistic people score higher on the TAS compared to NT, and are at greater risk of scoring as clinically alexithymic, it is important to note that not all autistic people captured in this review were alexityhmic. Of the papers examining cut-off rates, prevalence rates of alexithymia in the ASD groups ranged from 33.3% [3] to 63% [56], with a weighted mean prevalence rate of 49.93% compared to 4.89% in the NT groups. This highlights that, even at an upper estimate, nearly 40% of autistic people do not experience high levels of alexithymia, suggesting that although alexithymia may be common in autistic people, not everyone on the spectrum will experience alexithymia. This is consistent with the alexithymia hypothesis of ASD, and suggests that the nature and implications of co-occurring ASD and alexithymia warrants future research [8]. In particular, future research should examine the differences between individuals with co-occurring ASD and alexithymia, and ASD only. With research suggesting that increased rates of alexithymia in autism are associated with heightened anxiety and emotional difficulties compared to those with ASD only, it seems likely that the nearly 50% of autistic individuals with this co-occurring individiuals may have unique needs that require specific interventions [23]. On the basis of previous studies identifying alexithymia as a vulnerability factor for mental illness, particularly depressive disorders, future research should examine whether autistic individuals with alexithymia are indeed at a greater risk of developing mental health problems than those with ASD only [57]. Significantly, alexithymia has also been linked to a number of other negative health outcomes, including increased risk taking behaviour, poor physical health, and increased psychosomatic illness [58]. Further research is necessary to examine how co-occurring alexithymia in autism may result in unique needs, and how best these needs can be identified and met. Potential future directions could include screening autistic people for alexithymia to identify those at risk of associated health problems, particularly in mental health treatment settings where co-occurring alexithymia could be associated with poorer outcomes.

### 4.1 Limitations

One potential limitation of this study was that two different scales were included in the systematic review: the TAS-26, and the TAS-20. However, these are two highly similar measures: the TAS-20 was developed out of its earlier version, the TAS-26. The TAS-20 has a number of benefits compared to the TAS-26, including fewer items, and greater internal consistency, potentially reflecting why the majority of the studies captured in this review used the 20, rather than the 26 version ([29]; Kooiman, Spinhoven, & Trijsburg, 2003). However, the two measures significantly correlate with each other, even when controlling for depression, suggesting these are similar measures [30]. Moreover, steps were taken in the methodology to minimise the impact of using two different scales: standardised mean differences were used to compare mean scores, and only the TAS-20 was included in the prevalence analysis.

The variability of information reported across studies made direct comparison difficult, and particularly limited the ability of the meta-analysis to explore possible contributions towards the heterogenity of the findings: in particular, the meta-analysis was unable to explore potentially relevant factors including gender, depression and anxiety. Furthermore, the TAS was used in this study as a summary measure to explore alexithymia in ASD due to its widespread use in research. However, the limitations of the TAS, including the absence of items measuring fantazing, limited the ability of this review to further explore the nature of the alexithymia construct in this population. Previous research using additional alexithymia measures, such as the BVAQ, have highlighted that autistic people may have more difficulties with the cognitive aspects of alexithymia (for example identifying and verbalising emotions) rather than a lack of awareness of conscious experience [10]. However, the low number of studies using such an additional measure made it impossible to further explore these aspects in this review. Moreover, the TAS has only been validated for use in what has previously been described in the literature as “high-functioning” ASD, and consistently with this a large number of studies identified in this review specified that they recruited individuals with “high-functioning” ASD only [10]. The use of this self-report format may have excluded individuals with language or communication difficulties. Firstly, this means that the findings of this systematic review may not be generalisable across the ASD spectrum, but rather reflect those individuals specifically with no language or communication problems, and normal to high IQs. This is significant as a review of the literature suggested that there may in fact be more evidence for difficulties with emotional language in this specific ASD population, compared to those with additional intellectual disability or language problems [50]. Therefore, the question of whether alexithymia is heightened across the ASD spectrum requires further research using other, more appropriate measures.

## 5 Conclusions

By examining the use of the TAS in autistic people, this review demonstrated that up to 50% of autistic people experience co-occurring alexithymia: alexithymia appears to be heightened although not universal, in this population. This provides support for the alexithymia subgroup hypothesis of ASD, and for previous research indicating that emotional processing difficulties traditionally associated with ASD are in fact rooted in co-occurring alexithymia, rather than representing a core feature of ASD itself [6]. Further research is needed into the clinical implications, and the potential for targeted treatments, for this group. However, this review also highlighted methodological issues in the use of the TAS in ASD research that should be accounted for in future research. In particular, future studies exploring alexithymia in ASD should consider the use of additional measurements in tandemn with the TAS, and consider the role of the potentially confounding comorbidities of anxiety and depression in analysis.

## Declarations of interest

None.
