# Supplementary material for: Investigating alexithymia in autism: A systematic review and meta-analysis
Source: Eur Psychiatry. 2019 Jan;55:80–9. doi: 10.1016/j.eurpsy.2018.09.004 (PMC6331035; doi:10.1016/j.eurpsy.2018.09.004)
Supplement: Supplementary file 1 [file mmc1.docx]

|  | **Method** | | **Recruitment** | | | | | | | **Exposure (Alexiythmia) Measure** | | **Analysis** | | | **Results** | | | **Total quality score (max. 17)** |
| --- | --- | --- | --- | --- | --- | --- | --- | --- | --- | --- | --- | --- | --- | --- | --- | --- | --- | --- |
| **Paper** | **Addressed clearly focused issue?** | **Appropriate method?** | **ASD recruitment described?** | **ASD defined precisely?** | **ASD diagnoses confirmed?** | **Power or sample size calculation?** | **NT recruitment described?** | **NTs matched to ASD group?** | **Checked NT group for ASD?** | **TAS described?** | **Use of additional measures?** | **Confounding factors (comorbidities) measured?** | **Confounding factors accounted in analysis?** | **Analysis appropriate to design?** | **Do you believe the results?** | **Results applicable?** | **Results fit with other available evidence?** |  |
| Arellano et al. 2017 (1) | Y | Y | N | N | N | N | N | Age, IQ | N | Y | Y | N | N | Y | Y | Y | Y | 9 |
| Murray et al. 2017 (2) | Y | Y | Y | Y | Y | N | Y | Age, gender, verbal ability | Y | Y | N | N | N | Y | Y | Y | Y | 13 |
| Schaller & Rauh. 2017 (3) | Y | Y | Y | Y | Y | N | Y | Age, non-verbal intelligence | Y | Y | N | N | N | Y | Y | Y | Y | 13 |
| Hoffmann et al. 2016 (4) | Y | Y | Y | Y | Y | N | Y | IQ, gender | Y | Y | N | N | N | Y | Y | Y | Y | 13 |
| Milosavljevic et al. 2016 (5) | Y | Y | Y | Y | Y | N | Y | Age, gender | Y | Y | N | Depression and anxiety | N (insufficient power in TD group) | Y | Y | Y | Y | 14 |
| Patil et al. 2016 (6) | Y | Y | Y | Y | Y | N | N | Age, gender, level of education | N | Y | N | Depression | N | Y | Y | Y | Y | 12 |
| Krach et al. 2015 (7) | Y | Y | N | Y | Y | N | N | Age, gender, verbal intelligence | Y | Y | N | N | N | Y | Y | Y | Y | 11 |
| Berthoz et al. 2013 (8) | Y | Y | Y | Y | Y | N | Y | Age, gender (accounted for in analysis) | N | Y | Y | Depression and anxiety | Y | Y | Y | Y | Y | 15 |
| Schneider et al. 2013 (9) | Y | Y | Y | Y | Y | N | N | Age, gender, education | Y | Y | N | Depression. Psychiatric comorbidities also excluded in study design | N | Y | Y | Y | Y | 13 |
| Heaton et al. 2012 (10) | Y | Y | Y | Y | Y | N | Y | Age, gender, intelligence | Y | Y | N | N | N | Y | Y | Y | Y | 13 |
| Samson et al. 2012 (11) | Y | Y | Y | Y | Y | N | Y | Age, gender, level of education | Y | Y | N | N | N | Y | Y | Y | Y | 13 |
| Katsyri et al. 2008 (12) | Y | Y | Y | Y | Y | N | Y | Age, gender | Y | Y | N | N | N | Y | Y | Y | Y | 13 |
| Silani et al. 2008 (13) | Y | Y | N | Y | Y | N | N | Age, gender, IQ | Y | Y | Y | N | N | Y | Y | Y | Y | 12 |
| Lombardo et al. 2007 (14) | Y | Y | N | Y | Y | N | N | Age, gender | Y | Y | N | N | N | Y | Y | Y | Y | 11 |
| Tani et al. 2004 (15) | Y | Y | Y | Y | Y | N | Y | Age, gender, intelligence, body mass index (BMI) | Y | Y | N | Depression | N | Y | Y | Y | Y | 14 |

**Table A1: Quality assessment of included studies**
